# A Portable Device for the Generation of Drug-Loaded Three-Compartmental Fibers Containing Metronidazole and Iodine for Topical Application

**DOI:** 10.3390/pharmaceutics12040373

**Published:** 2020-04-18

**Authors:** Francis Brako, Chaojie Luo, Rupy Kaur Matharu, Lena Ciric, Anthony Harker, Mohan Edirisinghe, Duncan Q. M. Craig

**Affiliations:** 1 Medway School of Pharmacy, Universities of Kent and Greenwich, Chatham ME4 4TB, UK; f.brako@kent.ac.uk; 2 Department of Mechanical Engineering, University College London, Torrington Place, London WC1E 7JE, UK; chaojie.luo@ucl.ac.uk (C.L.); rupy.matharu.15@ucl.ac.uk (R.K.M.); 3 Department of Civil, Environmental & Geomatic Engineering, University College London, Chadwick Building, Gower Street, London WC1E 6BT, UK; l.ciric@ucl.ac.uk; 4 Department of Physics and Astronomy and London Centre for Nanotechnology, University College London, London WC1E 6BT, UK; a.harker@ucl.ac.uk; 5 School of Pharmacy, University College London, 29–39 Brunswick Square, London WC1N 1AX, UK

**Keywords:** trilayered fibers, portable electrospinning, wound dressing, combination therapy, compartmental drug delivery

## Abstract

The use of combination therapies for the treatment of a range of conditions is now well established, with the component drugs usually being delivered either as distinct medicaments or combination products that contain physical mixes of the two active ingredients. There is, however, a compelling argument for the development of compartmentalised systems whereby the release, stability and incorporation environment of the different drugs may be tailored. Here we outline the development of polymeric fine fiber systems whereby two drugs used for the treatment of wounds may be separately incorporated. Fibers were delivered using a newly developed handheld electrospinning device that allows treatment at the site of need. Crucially, the delivery system is portable and may be used for the administration of drug-loaded fibers directly into the wound in situ, thereby potentially allowing domiciliary or site-of-trauma administration. The three-layered fiber developed in this study has polyethylene glycol as the outermost layer, serving as a structural support for the inner layers. The inner layers comprised iodine complexed with polyvinylpyrrolidone (PVP) and metronidazole dispersed in polycaprolactone (PCL) as a slow release core. The systems were characterized in terms of structure and architecture using scanning electron microscopy, transmission electron microscopy, attenuated total reflection Fourier transform infrared spectroscopy and diffractometry. As antibacterial creams are still used for managing infected wounds, the performance of our trilayered fiber was studied in comparison with creams containing similar active drugs. Drug release was measured by UV analysis, while antimicrobial efficiency was measured using agar diffusion and suspension methods. It was found that the trilayered systems, averaging 3.16 µm in diameter, released more drug over the study period and were confirmed by the microbacterial studies to be more effective against *P. aeruginosa*, a bacterium commonly implicated in infected wounds. Overall, the portable system has been shown to be capable of not only incorporating the two drugs in distinct layers but also of delivering adequate amounts of drugs for a more effective antibacterial activity. The portability of the device and its ability to generate distinct layers of multiple active ingredients make it promising for further development for wound healing applications in terms of both practical applicability and antimicrobial efficacy.

## 1. Introduction

Chronic wounds remain a significant burden on the patient population and healthcare systems worldwide [1], with infection being the likeliest cause of delay in healing. Moreover, the formation of biofilms from the secretions of infecting bacteria, into which they become embedded, further inhibits healing. Indeed, biofilms are a significant factor in 70% of wounds that remain chronic [2]. One treatment approach is the application of antiseptics and antibiotics, preferably in combination, in order to simultaneously suppress biofilm formation [3] and prevent infection [4]. Administering more than one drug concurrently facilitates combinatorial selectivity, in which the different drugs involved can simultaneously inhibit several associated facets of a disease process. In addition, localisation allows for the efficient targeting of tissues, thereby maximising drug efficacy while also minimising toxicity via the control of the selectivity of the region of the body exposed to the therapeutic agent [5]. There is a need for wound management strategies that utilise a multifaceted approach to simultaneously target pathogeneses of the wound. For instance, diabetic foot ulcers (DFUs) are among the most difficult wounds to clinically manage, and it has long been established that anaerobes are typically associated with deeper and more severe infections in these kinds of ulcers [6]. Furthermore, as it is common to isolate several microbial species from infected wounds such as DFUs [7], an approach utilising combination therapies where multiple active agents are efficiently delivered simultaneously will be required for treating such wounds.

Combination therapies are nevertheless hampered by a lack of compartmentalisation between the associated drugs. Current clinical practice in wound care, for instance, involves the application of multiple and distinct delivery systems that lead to the variability of dose and poor control of local concentrations. A more elegant solution would be to deliver a compartmentalised system whereby, irrespective of the different physicochemical properties of the drugs, the possibility exists for tuning the release while also enhancing the stability and localisation of the different agents to maximise therapeutic benefits. Indeed, multi-compartmentalisation is regarded as a potentially crucial strategy for delivery as it offers the potential for the efficient delivery of diverse forms of active entities [8].

Our suggested method utilises an on-demand/need approach in which layered fibers are applied to wounds at the point of need, with each distinct layer containing a different therapeutic agent or a functionalised controlling layer. The middle layer has iodine stabilised in polyvinylpyrrolidone and intended as an antiseptic to sanitise the wound area, paving the way for a more effective anti-bacterial action from metronidazole contained in slowly degrading polycaprolactone making up the core of this unique fiber system. Iodine, in previous studies, has been shown to effectively inhibit the formation and growth of biofilms [3], thus informing our decision to combine it with metronidazole for a multi-targeted approach to wound management. The compartmental encapsulation of drugs in distinct layers of polymeric fibrous systems has several potential advantages. Specifically, the fibrous mesh has a high surface area and allows fluid permeation, while the layered structure within the fibers allows the separation of drugs into physically distinct environments with the potential for tunable controlled release. In addition, the physical flexibility and versatility of the macroscopic mesh structure allow lesions of irregular shapes to be filled neatly [9,10].

These potential advantages of combination therapies have led to a significant increase in research into compartmentalised structures as patient-specific drug delivery systems [11,12]. However, several limitations hamper their practical applicability in a clinical setting. Despite recent advances in production technologies such as multi-layer electrospinning and significant improvements in scale-up, the production of multi-layered drug-loaded fibers requires a skilled worker operating expensive, bulky and essentially immobile bench-top apparatus in a specialised laboratory or factory environment. Furthermore, the highly delicate nature of the fibers produced renders it difficult to preserve structural integrity during packaging, transport and administration from the point of manufacture to the point of use.

It is therefore therapeutically highly advantageous to develop a portable and inexpensive device that may produce multi-layered therapeutic materials in situ at the point of trauma. While the design of a portable electrospinning device has been reported by several research groups [13,14,15], none of these is capable of producing multi-layered structures that can be useful for developing multi-compartment systems for the efficient simultaneous delivery of different drugs. Here, we describe a simple-to-operate, miniaturised portable device for generating trilayered fine structures at the point-of-use. As a proof-of-concept to explore the use of a trilayered fibrous system, we develop a new, improved combination therapy consisting of iodine and metronidazole (MTZ) for wound dressing applications. We demonstrate the potential clinical efficacy of this approach using drug release and antimicrobial studies; more specifically, metronidazole (MTZ) is an antibiotic agent widely recognised as a standard for the elimination of anaerobic bacteria [3]. Moreover, formulations combining the antibacterial MTZ with the antiseptic iodine have been found to exhibit promising synergistic wound healing properties [16]. The MTZ-iodine formulations are typically presented as ointments in which the drugs are simply mixed as physical combinations [17]. Here, we load the active ingredients into different polymeric compartments in a trilayered fiber using the portable device. More specifically, MTZ dispersed in a biodegradable polymer, polycaprolactone (PCL), makes up the innermost core of the trilayered fiber. The middle layer contains iodine stabilised in polyvinylpyrrolidone (PVP), the former acting as an antimicrobial agent to rapidly sanitise the wound upon application. Finally, the outermost layer is made of a polyethylene glycol (PEG) polymer as the shell fabric, acting to provide physical robustness and moisture regulation as well as a wound barrier to prevent contamination.

The potential advantages of the new approach are manifold. First, by keeping each ingredient in a separate compartment the performance and stability of the formulation are enhanced as interactions (should they exist) between the drugs are minimised prior to the onset of action. Secondly, the core-shell fibrous structure provides a controlled drug release while acting as an effective physical protection and dressing for the wound. Thirdly, polymeric systems have been demonstrated to reduce irritation and toxicity associated with antiseptics such as iodophores when used in wound care [18]. Lastly, the portable production method allows highly specific systems to be tailored/personalised and delivered on-site for a desired therapeutic outcome while eliminating the need for the packaging and transport of the delicate product to the site of use, thereby further preserving the drug and dosage form integrity. Here, we explore the manufacturing feasibility, structural architecture and potential therapeutic efficacy of the portable trilayered system so as to establish the applicability of the approach to both the treatment of wound infections and, more broadly, the treatment of in situ conditions under a range of settings.

## 2. Materials and Methods

### 2.1. Materials

Metronidazole (MTZ, analytical standard, Mw = 171.15 g/mol), polyvinylpyrrolidone-iodine complex (PVP-I, European Pharmacopoeia Reference Standard with average Mw = 365 g/mol), high molecular weight polyvinylpyrrolidone (PVP, Mw = 360,000 g/mol), polycaprolactone (PCL, Mw = 80,000 g/mol), polyethylene glycol (PEG, BioUltra, Mw = 35,000 g/mol), dichloromethane (DCM), dimethylformamide (DMF) and ethanol (EtOH) were purchased from Sigma Aldrich (Poole, Dorset, UK) and used as received. 

MTZ and metronidazole-iodine (MTZ-I) in aqueous cream BP (British Pharmacopoeia) base were prepared as follows: 10 g of the MTZ cream comprised of 0.1 g of MTZ (batch number MKBZ3056V, Sigma Aldrich), 3 g of emulsifying ointment (batch number NR 10043, Pinewood Healthcare, Dublin, Ireland), 0.1 g of phenoxyethanol (batch number STBD 3014V, Sigma Aldrich) and made up to the target weight with purified water. The MTZ-I cream contained the same ingredients as the MTZ cream with the addition of 0.5 g PVP-I, amounting to 0.5% elemental Iodine (equivalent to a 2:1 MTZ: PVP-I weight ratio). Lysogeny broth (LB) and LIVE/DEADTM BacLightTM Bacterial Viability and Counting Kits were purchased from Thermo Fisher Scientific (Waltham, MA, USA), whilst LB agar was purchased from Sigma Aldrich. 

### 2.2. Solution Preparation and Characterization 

To prepare the trilayered electrospun fibers, different polymer or polymer/drug solutions were prepared, selecting materials known to enable optimised encapsulation and stable trilayered electrospinning as well as to fulfil the objective of developing a system which combined MTZ and Iodine for a synergistic antimicrobial outcome. Materials and parameters used in developing this system are shown in Table 1.

The physical properties of the three solutions used for the three layers of the fibers were characterised. The surface tension was measured with a Kruss Tensiometer K9 (Hamburg, Germany) using the standard Wilhelmy’s plate method. The electrical conductivity was measured using a conductivity meter (Jenway 3540, Bibby Scientific, Stone, Staffordshire, UK). The viscosity was measured with a digital rotational viscometer (Brookfield DV-111, Harlow, Essex, UK) using spindle number 18 at a shear stress of 3.5 Pa. All procedures were repeated five times, and the mean value of the readings were recorded. The viscometer was calibrated automatically by following prompts to run a complete cycle without the spindle in place. The tensiometer was calibrated by taking the surface tension of distilled water at room temperature and comparing it with the literature value. The conductivity meter was calibrated by immersing the probe in the conductivity standard (Bibby Scientific, Staffordshire, UK), and the meter was adjusted until a reading of 1194 µS cm^−1^ at 22 °C was obtained.

### 2.3. Portable Trilayered Electrohydrodynamic Apparatus and Processing Conditions

**Figure 1:**
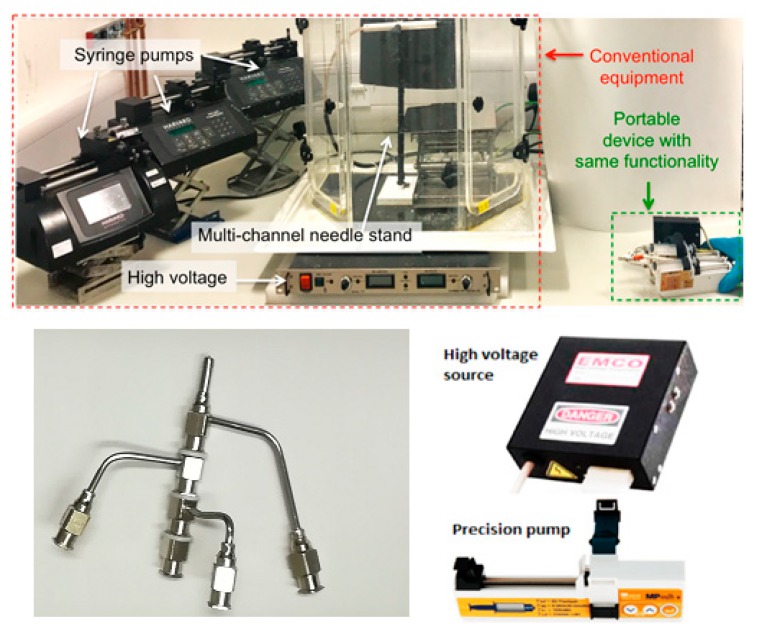
Above: Photograph showing a size comparison between the old conventional large, bench-top setup and the new three-compartment portable electrohydrodynamic device held by a researcher. Below: A photograph of the multiple needle system used to generate three-layered fibers and the high voltage source and precision pump used in developing the device.

The portable trilayered electrohydrodynamic apparatus is shown in Figure 1. Additional photographs and a schematic diagram is shown in the Supplementary Information (Section S1, Figure S1). The three different solutions were each loaded in a 10 mL syringe attached to a stainless-steel concentric needle system with multiple inlets and outlets. The concentric needle system is an additive system that can be changed between single-layer, two-layer, trilayer and quad-layer depending on the number of layers required for the products; in this work, the system is used in the trilayer mode. Fibers were generated using the positive polarity of the voltage supply and collected on a grounded aluminum plate. Three miniature high-precision micro-syringe pumps (Micrel mph+, Inspiration Healthcare Limited, UK) were used, each supplying one of the three solutions through the concentric needle system, charged by a miniature high voltage supply able to generate up to 33 kV at 10 W (EMCO 4330+, XP Power, UK). The solutions were electrospun at a positive voltage of 17 kV, a working distance (between the needle exit and the collector) of 15 cm, and flow rates of 2, 4 and 7 mL h^−1^ for the core, middle and shell solutions, respectively (Table 1). All electrospinning procedures were carried out under an ambient temperature, humidity and pressure (22 °C, 1 atmospheric pressure, 40–50% relative humidity), as described in our previous work [19].

### 2.4. Fiber Characterisation 

The surface morphology and diameter of the fibers were studied using a Quanta 200F field emission scanning electron microscope (Thermo Fisher Scientific). Prior to observation, each sample was coated with gold using a Quorum Q150T turbo-pumped sputter coater (Quorum Technologies, Lewes, East Sussex, UK) for 90 s. The average fiber diameter was determined using Image-Pro Plus software (Media Cybernetics, Inc., Rockville, MD, USA) and was based on at least 100 measurements from the scanning electron microscope (SEM) images. In order to confirm that the fibers had three distinct layers, transmission electron microscope (TEM) imaging of fibers collected on carbon-coated grids was conducted using TEM CM 120 Biotwin (Thermo Fisher Scientific), operating at an accelerating voltage of 100 kV.

To verify the composition of the fibers, attenuated total reflection Fourier transform infrared spectroscopy (ATR–FTIR) was carried out using a Vertex 90 spectrometer (Bruker, Coventry, West Midlands, UK). The measurements were interpreted using OPUS Viewer version 6.5 software. Each sample was scanned 32 times at a resolution of 2 cm^−1^. To investigate the solid-state of the drugs entrapped in the multi-layered fibers, samples were analysed using a D/Max-BR diffractometer (RigaKu, Tokyo, Japan) with Cu Kα radiation. Analyses were conducted at 40 mV and 30 mA over the 2θ range of 5°–80° at a rate of 2° min^−1^. The data obtained were converted to diffractograms and evaluated using OriginPro 7.0 software (OriginLab Corporation, Northampton, MA, USA).

### 2.5. Drug Release Study

To measure and compare the amount of drug released respectively from the fiber and cream formulations, we used a system utilising a dialysis membrane as a means of filtration to minimise the extent of other materials from either the trilayered fibers or creams interfering with the amount of MTZ sampled for the UV analysis. 50 mg of the drug-loaded sample was first enclosed in a 5 mL dialysis membrane with a molecular weight cut-off of 8000 g mol^−1^. The sample was then immersed in a 45 mL vessel (sampling chamber). A physiological temperature of 37 °C and a continuous agitation at 80 rpm were maintained throughout the release study using a benchtop incubation shaker (Sciquip, Newtown, Shropshire, UK). The total duration of the study was five days. Samples of 3 mL were taken at various intervals from the vessel. A 3 mL blank medium was added after each sampling to maintain a constant total volume.

An ultraviolet-visible (UV–Vis) spectroscopy (Jenway 6305 UV/Visible spectrophotometer, Bibby Scientific, Stone Staffordshire, UK) operating at 322 nm was used to quantify the amount of drug present as a function of time. To determine the total amount of drug entrapped during fiber formation, 1 mg of the MTZ-loaded trilayered fibers was dissolved completely in 10 mL of dichloromethane, followed by measuring the solution’s absorbance.

### 2.6. Antimicrobial Activity

Two microbial inhibition assays were carried out to compare the antimicrobial activity of the prepared trilayered fibers and MTZ-I/MTZ cream formulations. The first assay assessed the area of inhibition on an agar plate, while the second assay assessed the 24-h bactericidal results in cell suspensions analysed by flow cytometry. *Pseudomonas aeruginosa* NCTC 12903 (Public Health England) was chosen as the model microorganism to assess the antibacterial properties of the prepared trilayered fibers and MTZ-I/MTZ cream formulations. *P. aeruginosa* is one of the most common bacteria isolated from chronic wounds, typically resident in the deepest region of the wound bed [20]. Furthermore, *P. aeruginosa* is well known for its high virulence, causing severe morbidity by releasing proteases and cytotoxic substances in chronic wound environments and thereby seriously impeding wound healing [21]. *P. aeruginosa* NCTC 12903 is isolated from blood cultures and is commonly used for susceptibility testing. *P. aeruginosa* NCTC 12903 was propagated as per the manufacturer’s instructions and stored at −80 °C in a MicrobackTM.

#### 2.6.1. Agar Diffusion Assay

A single colony of *P. aeruginosa* NCTC 12903 was suspended in 100 µL of sterile deionised water and transferred to an LB agar plate. A plastic L-shaped spreader was used to spread the bacteria over the plate. The plate was allowed to air dry. 1 g of the fibers and cream were placed in the centre of the plate and had an area coating of approximately 1 cm in diameter (concentration of 10 wt %). After application, it was ensured that the fibers had made complete contact with the agar surface. The plates were incubated for 24 h at 37 °C. The assay was performed in triplicate for each treatment method. The antibacterial activity was assessed by the diameter of the growth inhibition zone. Photographs of agar plates and inhibition zones are shown in the Supplementary Information (Section S3, Figure S3).

#### 2.6.2. Suspension Assay

A method previously used for assessing antimicrobial activity [22] was adapted for this study. A single colony of *P. aeruginosa* was suspended in 30 mL of sterile LB broth. The suspension was incubated at 37 °C and 150 rpm until it reached its mid-exponential phase (OD600 0.4, equivalent to 8 Log10 CFU/mL). A 1:100 dilution of the bacterial suspension was inoculated into sterile LB broth. The fibers and cream were respectively added to the bacterial suspensions at 10 wt % and incubated for 24 h at 37 °C and 150 rpm. Flow cytometry was used in conjunction with the LIVE/DEAD BacLight Bacterial Viability assay to enumerate the number of live and dead cells in the suspension post-incubation. This method relies on the use of fluorescent stains, SYTO^®^ 9 and propidium iodide (PI) (both from Thermo Fisher Scientific). SYTO^®^ 9 is a green fluorescent nucleic dye which is able to penetrate both live and dead cells, whilst PI is a red fluorescent intercalating stain which can only penetrate cells with damaged membranes (non-viable cells) to displace the SYTO^®^9 and which results in a red colouration. A stock solution of PI and SYTO^®^ 9 was prepared according to the manufacturer’s instructions. 180 µL of the stock staining solution was added to 20 µL of diluted sample and incubated at room temperature in the dark for 15 min.

After incubation, cells were acquired using a calibrated Guava easyCyte^®^ flow cytometer and InCyte software (guavaSoft 3.1.1) (Luminex Corporation, Austin, TX, USA). Gates were set up using positive (media and bacteria only), negative (media only) and fluorescent minus one control (single-stained positive controls). 50,000 events were collected, and the bacteria acquisition gates were determined using forward scatter and side scatter channels to eliminate background noise and debris from the sample. The gated population of bacteria was then analysed using fluorescent channels. FlowJo was used to gate the live and dead bacterial cell counts, where the proportions of live and dead bacteria cells were calculated. All experiments were repeated at least three times.

## 3. Results

### 3.1. Portable Multi-Layer Electrospinning Device

The core-shell concentric electrospinning device could be a useful innovation in and of itself, in that this portable assembly can be readily adapted to generate single-layer, core-shell layer, three-layer and four-layer products, depending on the number of compartments required. The components for the portable multi-layer electrospinning device are off-the-shelf and simple to assemble; the assembly of the miniature high voltage (HV) supply and precision syringe micro-pumps are less expensive but as effective as their heavy, voluminous and practically immobile bench-top counterparts. The portable HV supply (adapted from EMCO 4330+, XP Power) can produce 0–33 kV at 10 W, a wider range than a bench-top HV supply which typically supplies 0–30 kV. The mini HV unit weighs less than 0.7 kg at a volume of 0.28 m^3^, and costs < £800. This is 95 times smaller, 10 times lighter and 3 times cheaper than a traditional HV unit (~7 kg, 27 m^3^, £2–5k).

The high precision micro-pumps (Micrel MP mlh+, Micrel Medical Devices SA, Koropi, Athens, Greece) are compact, lightweight and affordable (each at 0.15 m^3^ (volume), 0.2 kg (weight), when inclusive of 6 AAA batteries, and retailing at £1k). The battery-operation can sustain up to 600 h of continuous infusion at a high precision of 0.1 mL h^−1^. These micro-pumps are as effective as conventional syringe pumps for electrospinning but are at least 50 times smaller, 10 times lighter and 4 times cheaper (e.g., conventional precision syringe pumps are ~7.5 m^3^, 2 kg and retail at £2–4k). The apparatus is easy to operate and can be hand-held for the direct generation of structures (both particles and fibers) at the point-of-use (Figure 1). Previously, we demonstrated how this miniaturised device could be used in the manufacture of a system containing silver nanoparticles as antibacterial agents to be applied as a wound dressing [17]. This study represents, however, the first time trilayered electrospinning has been achieved using a truly miniaturised setup.

### 3.2. Solution Compositions for Optimal Trilayered Fiber Formation

**Figure 2:**
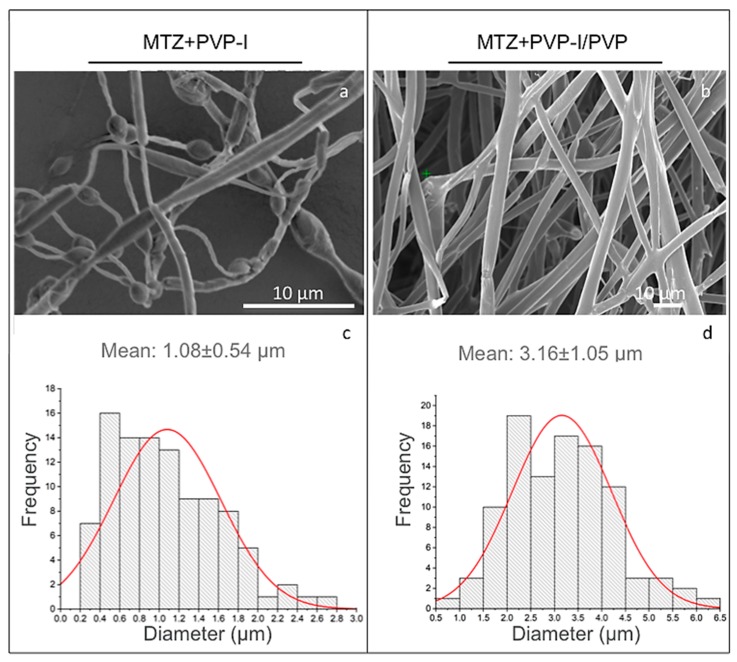
(**a**,**b**) SEM images of the fibers, and (**c**,**d**) the corresponding fiber diameter distribution profiles. Left panel: beaded trilayered fibers, generated with 10 wt % PVP-I in the middle layer. Right panel: smooth trilayered fibers, generated with 10 wt % PVP-I/PVP (1:1 w/w) in the middle layer.

To generate trilayered fibers with distinct compartments containing MTZ and iodine under optimal processing conditions, we first characterised the physical properties of each solution used for the trilayered co-flow (Table 2). Trilayered fibers generated from 10 wt % of PVP-I solutions were found to be beaded, with an average diameter of 1.08 ± 0.54 µm (Figure 2a,c). The bead-on-string morphology is undesirable because it compromises the compartmental integrity of the trilayered fibers. It is well known that increasing the solution viscosity leads to a reduction in the undesirable bead-on-string morphology [23]; the viscosity of 10 wt % of PVP-I solutions at 4.1 ± 0.3 mPa·s was too low to enable a sufficient molecular chain entanglement for bead-free fiber formation (Table 2). By mixing the PVP-I complex with a high molecular weight PVP (Mw at 360,000 g/mol, Table 1), the resultant PVP-I/PVP solution had a higher viscosity of 78.6 ± 1.2 mPa·s and enabled bead-free fiber formation (Table 2, Figure 2b). The smooth trilayered fibers had an average diameter of 3.16 ± 1.05 µm (Figure 2d). The increased fiber diameter is attributed to both the increased viscosity as well as the lower electrical conductivity of the PVP-I/PVP solution in comparison to the pure PVP-I solution used in forming the beaded fibers [24]. The electrical conductivity of the pure PVP-I solution was 340 ± 4 µS m^−1^, which is nearly threefold higher than that of the PVP-I/PVP solutions at 121 ± 0.5 µS m^−1^. A decrease in solution electrical conductivity is known to generate larger fiber diameters in electrospinning [25]; hence, these results are compatible with the existing knowledge base.

The bead-free, smooth continuous fibers comprising PCL + MTZ, PVP-I/PVP (1:1) and PEG, henceforth referred to as trilayered fibers, were used for the subsequent analysis in this study.

### 3.3. Analyses of the Trilayered Fiber Compositions

#### 3.3.1. Structural Features

**Figure 3:**
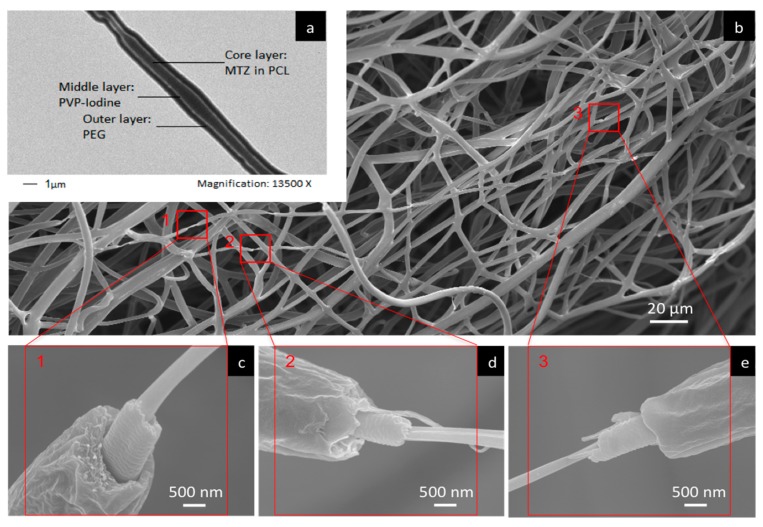
(**a**) TEM and (**b**–**e**) SEM images showing the three-layer compartmental structure of trilayered fibers, generated with 10 wt % PVP-I/PVP (1:1 w/w) in the middle layer.

TEM and SEM analyses were performed to confirm the multi-layered structure (Figure 3). The three distinct layers were clearly observed under TEM (Figure 3a), confirming the three-layer core-shell structure of the fibers. As shown in the TEM image, the core layer corresponds to the MTZ-PCL centre, the middle layer corresponds to the PVP-I complex, and the outermost layer corresponds to the PEG shell of the fiber. While an examination of the fibers by SEM can typically only reveal the surface morphology (Figure 3b), the images shown in Figure 3c–e nevertheless allow the three layers to be visualised for those fibers that demonstrate differential extensions of the layers.

#### 3.3.2. Compositional Features

**Figure 4:**
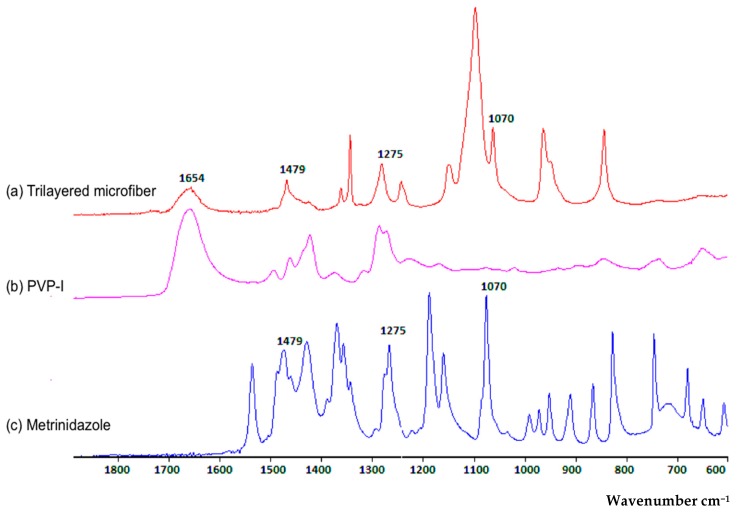
FTIR spectra of the (**a**) trilayered fiber; (**b**,**c**) the active components: (**b**) PVP-I and (**c**) metronidazole.

The FTIR spectra of MTZ and iodine-loaded trilayered fibers were compared to those of pure MTZ and PVP-I, to confirm the entrapment of the active ingredients in the fibers (Figure 4). Free iodine interacts with the negative end of the C=O dipole of PVP molecules, forming a complex with the amide functional group and is identified as a characteristic peak around 1654 cm^−1^ [26,27]. This characteristic peak was seen in the PVP-I spectrum as well as the fibers, confirming the presence of iodine. Peaks occurring at 1479, 1275, 1070 and 870 cm^−1^ representing the N=O, C–O, C–N and C–NO_2_ stretching are characteristic of MTZ [28,29]. These characteristic peaks were observed in both the pure MTZ compound and the generated fibers, confirming the entrapment of MTZ in the fibers.

**Figure 5:**
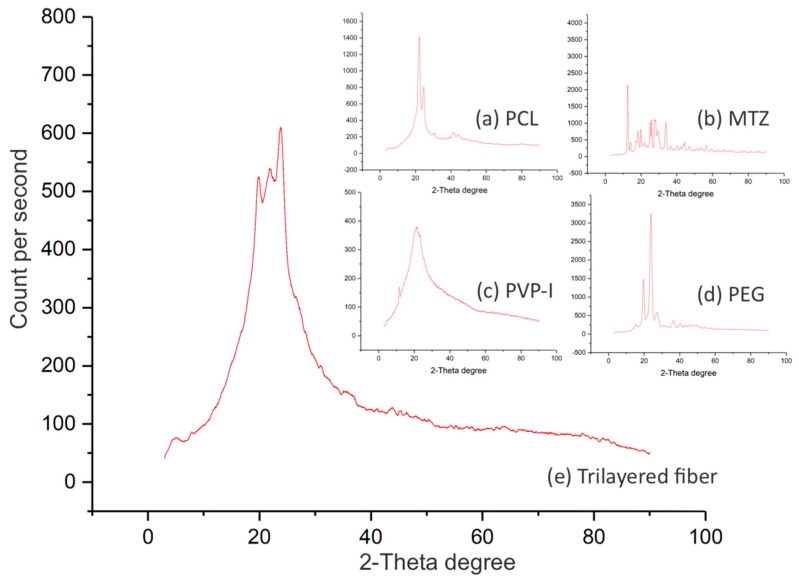
XRD patterns for (**a**) PCL, (**b**) MTZ, (**c**) PVP-I, (**d**) PEG and (**e**) fiber. XRD patterns of the PCL, MTZ and PEG samples show peaks characteristic of their crystalline state, whereas the PVP-I and the trilayered fiber samples are largely amorphous.

Understanding the solid-state of the active ingredients in the drug-carrying fibers, whether it be amorphous, crystalline or a mixture of both, can help inform the likely performance and storage behaviour of such formulations [30]. The XRD spectra of the fibers were compared to those of the individual active ingredients to determine the crystalline state of the drug-loaded fibers. The differences between the spectra reflect the extent of change that the solid-state of the starting material has undergone post-processing. Figure 5 shows that PCL, MTZ and PEG are in their crystalline state (Figure 5a–c), while the PVP-I is largely amorphous (Figure 5d).

The drug-loaded fibers containing the aforementioned materials were found to be part-crystalline and part-amorphous (Figure 5e), almost certainly reflecting the crystalline components of the polymers (PCL and PEG). However, the extent of crystallinity of the polymers in such a complex architecture is difficult to ascertain, given the known propensity of electrospinning to elicit polymer chain reorganisation during electrospinning [31]. While the presence of crystalline MTZ as a minor component cannot be completely precluded on the basis of this data, the absence of any evidence for major MTZ peaks at angles where the polymer response was low (and hence unlikely to hide the drug response) would support drug amorphisation. MTZ is sparingly soluble in aqueous systems, and hence molecular dispersion in the fiber would promote dissolution via the absence of a crystal lattice, in turn potentially aiding bioavailability.

### 3.4. Drug Release Study

MTZ is topically administered via creams, gels or lotions in strengths typically ranging between 0.75 and 1 wt % [15]. With this as a guide, the concentrations and flow rates of the core-middle-shell solutions during trilayered electrospinning were optimised (Table 1) to enable a final encapsulation concentration of 1 wt % MTZ within the total volume of the core-shell fiber.

**Figure 6:**
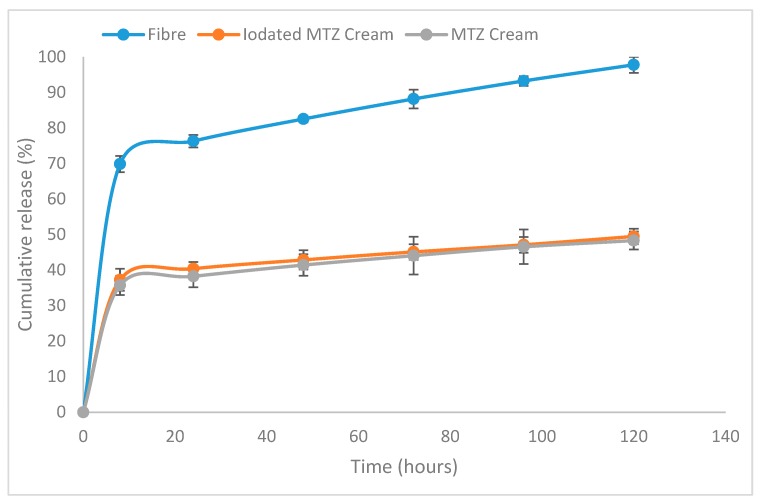
Cumulative percentage of drug (MTZ and iodated MTZ) released from the trilayered fiber systems and cream formulations obtained during the study over a five-day period.

Drug release from the trilayered fibers was compared to those from creams containing similar quantities of MTZ. Antibiotic creams are currently routinely applied as part of wound dressings, with the hope of releasing the active drug contained therein over the period during which the wound dressing is in place, usually over several days [32,33,34]. While the intended purpose of the fibers (treatment of wound infection and promotion of healing via tailored release of active drug, moisture control and provision of a physical barrier to prevent further contamination all in one) is distinct from that of antibacterial creams for wounds, there is merit in comparing the new formulation to an existing and established delivery system, particularly in terms of looking at the longer-term supply of adequate amounts of active drugs than would typically be required from a cream. The drug release study from the fibrous formulation and two cream formulations (MTZ with and without iodine) is shown in Figure 6. To compare the release of drug from the trilayered fibers and creams, a setup comprising the delivery system enclosed within a dialysis membrane in a volume of 5 mL and further immersed in a liquid with a volume of 45 mL (outer chamber) was used, from which sampling was made from the outer chamber to determine the amount of drug released. This was to ensure that the delivery systems being studied were totally immersed within the dissolution media, as both systems had tendencies to float and disrupt the continuous release of the drug into the media. In this two-chamber setup, sampling for analysing the drug release was possible from the outer chamber, but as the drug was released into the inner chamber enclosed by a dialysis membrane it was necessary to establish the relationship between the sampled drug and the actual amount of drug released into the dialysis chamber. For a more robust comparison between the drug released from creams and the trilayered fibers, it was necessary to establish that, regardless of the difference in the diffusion coefficient (which is certain to be the case for creams and fibers), the amount of drug sampled from the outer chamber for analysis maintains its relationship with the actual amount of drug released within the dialysis membrane.

To determine this relationship, some assumptions and the modelling of the experimental data from the study were made. These were essential to establish an accurate comparison of drug release from the trilayered fibers and creams. The mathematical modelling of the experimental data is presented as Supplementary Information (Section S2, Figure S2).

### 3.5. Release from the Trilayered Fiber System and Creams

As it has been established (in the Supplementary Information, Section S1) that the sampled drug profiles reflect the profiles of the actual drug released, regardless of the system being assessed, the amounts of drug sampled from the fiber and cream systems were compared. From the fiber system, 97.8 ± 2.3% of MTZ had been released after five days. In contrast, only 48.5 ± 2.2% and 49.5 ± 2.7% had been released from the MTZ and MTZ-iodine cream formulations, although all systems being compared were formulated to contain the same amount of MTZ. In addition, the inclusion of iodine in the cream formulations was not found to influence the MTZ release significantly. It has been reported for creams that only the fraction of the MTZ present in the external aqueous phase would be able to diffuse out for therapeutic activity [35,36]. On the other hand, MTZ as a solid dispersion in a polymer matrix in the trilayered fiber is most likely released via drug diffusion and matrix erosion [37], a process significantly facilitated by the increased surface area to volume ratio acquired by the compartmental, fiber delivery format. Suboptimal concentrations of antibiotics at topical infection sites have been confirmed to confer a survival advantage of bacteria leading to antimicrobial resistance and ultimately clinical failures [38]. Having a solid dispersion of MTZ in a polymeric matrix ensured the release of more antibiotics from the fibers, as seen in the release study, and this was confirmed to offer better antibacterial action against the test organisms used in our antimicrobial assay, discussed in Section 3.5. The examination of the first 8 h of release indicated that 69.9 ± 2.3% of MTZ in the compartmentalised fiber system had been released compared to 37.3 ± 3.2% and 35.7 ± 2.8% of the MTZ-I and MTZ cream formulations, respectively. Thus, the three-layered fiber system, unlike the cream formulations, appears to deliver more of the active ingredient within the specified time.

### 3.6. Antimicrobial Assays

*P. aeruginosa*, a common Gram-negative rod-shaped bacterium known for its resistance to antimicrobials [39], was selected as the test microorganism to assess the antibacterial activity of MTZ-loaded fibers compared with MTZ and MTZ-I creams. Furthermore, biofilms secreted by *P. aeruginosa* are commonly associated with difficult-to-heal chronic wounds [40], and hence this organism provides a highly relevant model for the novel fiber delivery system. Previous research has shown MTZ to be an effective antibiotic against resistant strains of *P. aeruginosa* when used in combination therapies [41].

Two antimicrobial assays were employed: (1) an agar diffusion assay to investigate the performance on a surface, thereby mimicking topical delivery (Supplementary Information, Section S3.1, Figure S3), and (2) a bacterial cell suspension approach to investigate the antimicrobial performance of the fibers when immersed in liquid media, hence mimicking anaerobic conditions.

Flow cytometry was used to give a rapid, comprehensive and quantifiable overview of the bacterial population post-treatment (Supplementary Information, Section S3.2, Figures S4 and S5). Previous literature has shown flow cytometry to provide a more accurate quantitative measurement of cell viability when compared to plate count estimates [22].

**Figure 7:**
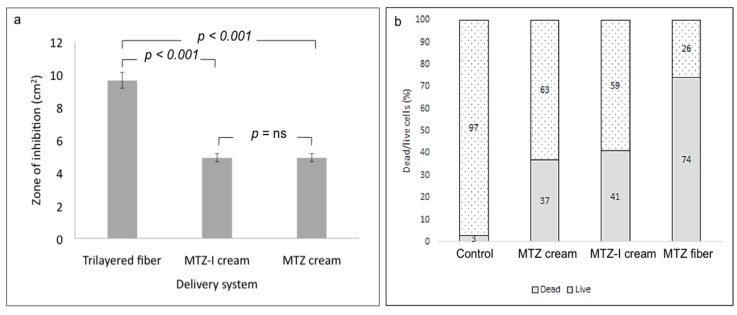
(**a**) Growth inhibition zone area of P. aeruginosa following 24-h incubation on LB agar plates with various drug formulations. (**b**) Dead/live cell count by flow cytometry of cell suspensions incubated for 24 h with drug-loaded fiber and cream samples.

In both assays, the trilayered fibrous formulation demonstrated a more potent elimination of *P. aeruginosa* cells than the MTZ and MTZ-I creams did. The antimicrobial effect was due to the presence of MTZ, as the drug-free fiber controls showed no antimicrobial action while the formulations containing iodine in addition to MTZ did not result in significantly higher cell death rates (Figure 7). More specifically, in the agar diffusion assay, the trilayered fibers inhibited a circular area with an average diameter of 35 ± 1.4 mm, whereas the MTZ and MTZ-I formulations resulted in growth inhibition zones with average diameters of 25 ± 1 mm and 25 ± 2 mm, respectively (Figure 7a shows the corresponding zone areas).

Additionally, a 74% cell death was noted after exposure to the trilayered fiber samples in suspension, whereas a rate of 37% and 41% cell death, respectively, was noted after exposure to MTZ and MTZ-I cream formulations over the same period of incubation (Figure 7b). The more significant cell death rate found in samples with the fibers is attributed to the better release of MTZ from the trilayered compartmental system, which is also confirmed by the drug release study in Section 3.4. The enhanced release from the fiber system enabled a better availability and stronger bactericidal action, compared to the creams containing similar quantities of MTZ. The multi-layered fiber structure allowed the release of MTZ and iodine from different compartments and facilitated the antibacterial actions of the active ingredient. The inherent properties, mainly a high surface-to-volume ratio and surface porosity of fibers, expedited the release of active ingredient for antibacterial activity, whereas the limited activity in the cream formulations are attributed to the fact that the non-compartmental system only makes a small fraction of the active ingredients available for antibacterial action.

## 4. Discussion

Re-engineering an electrospinning device to be more accessible and practically useful for delivering multiple active ingredients simultaneously is an intervention with a tremendous potential for improving the management of difficult chronic wounds. One such challenging clinical condition that stands to benefit from such an innovation is diabetic foot ulcers (DFU), given the need to deliver multiple active agents to simultaneously target the various underlying causes of such wounds. Presently, the gold standard for treating difficult infected chronic wounds where vascularisation is impaired includes debridement, treating the infections, the application of growth factors and modulators, revascularisation and off-loading the ulcer [42]. In treating infections and applying other interventions such as growth factors, agents are typically applied separately, and wound dressings capable of delivering multiple agents simultaneously are yet to be used. Electrospinning systems that could potentially deliver such interventions are currently in preparatory stages [43].

Therefore, developing a multi-layered system that clearly isolates active ingredients from each other until required for activity shows tremendous potential for improving the healing process of such difficult-to-heal wounds. Having demonstrated that our portable multi-compartment fiber generating system is capable of releasing more antibiotics in comparison to creams for a superior antibacterial activity points to the possibility of further developing this portable electrospinning device into an efficient and cost-effective means of managing difficult wounds in the near future.

## 5. Conclusions

In summary, this work presents three novel findings: (1) the point-of-need generation of trilayered fibers containing compartmentalised drugs for in situ wound treatment; (2) a simple-to-operate, miniaturised portable device for the electrospinning of multi-layered core-shell structures at the point-of-use; (3) the delivery of multiple active drugs for a possible combination therapy using a core-shell fiber configuration. The drug-loaded fibers were characterised by scanning electron microscopy, transmission electron microscopy, attenuated total reflection Fourier transform infrared spectroscopy and X-ray diffraction. In addition, the trilayered fibers demonstrated a superior antibacterial activity in comparison with the non-compartmental cream systems. This work paves the way for applying multiple therapeutic agents simultaneously in a compartmental format at the point-of-need and strengthens the prospects of having more efficient and cost-effective means of managing difficult wounds in the foreseeable future.

## Figures and Tables

**Table 1:** Solution composition and corresponding flow rates used in the various sections of our multiple concentric needle systems for trilayered electrospinning.

Fiber Layer	Flow Rate (mL h^−1^)	Material	Solvent
Core layer	2	4 wt % MTZ + 12 wt % PCL	4:1 v/v DCM: DMF
Middle layer	4	10 wt % PVP-I or 10 wt % PVP-I/PVP (1:1 w/w)	EtOH
Shell layer	7	25 wt % PEG	DCM

**Table 2:** Physical properties of the electrospinning solutions used to generate trilayered fibers.

Solution	Concentration (wt %)	Viscosity (mPa·s)	Surface Tension (mNm^−1^)	Conductivity (µSm^−1^)
PCL + MTZ	12 + 4	1355.0 ± 6.4	40.2 ± 0.6	5.37 ± 0.04
PEG	25	1605.0 ± 5.0	39.0 ± 0.9	2.90 ± 0.02
PVP-I	10	4.1 ± 0.3	24.6 ± 0.3	340.00 ± 4.00
PVP + PVP-I 1:1 w/w	10	78.6 ± 1.2	27.8 ± 0.3	121.00 ± 0.50

## Supplementary Materials

The following are available online at https://www.mdpi.com/1999-4923/12/4/373/s1. Figure S1: (a) Assembling various components of the portable electrospinning device; (b) the device in operation, producing yellowish metronidazole/iodine loaded fibers; and (c) a schematic representation of the device setup. Figure S2. (a) Fractional release from the delivery systems (blue) and into the outer liquid (orange) as a function of time for scenarios with varying diffusion coefficients from the delivery system (*D_p_*) and through the dialysis membrane (*D_m_*). (b) Cumulative percentage of drug released from the trilayered fibersystems and cream formulations obtained during the study over a five-day period. Figure S3. Photographs of the results showing the growth inhibition of *P. aeruginosa* following the 24-h incubation of the bacterial cells on LB agar plates with: (a) MTZ cream, (b) MTZ-I cream, and (c) triaxial fibers. Figure S4. A schematic representation of cell sorting using flow cytometry [Image source: Sari Sabban ©2011 / CC BY-SA (https://creativecommons.org/licenses/by-sa/3.0)]. Figure S5. Dead/live cell count by flow cytometry of cell suspensions incubated for 24 h for (a) control (without cream or fiber) 4% dead/96% live, (b) plain fibers without drug 3% dead/97% live, (c) drug-loaded cream 59% dead/41% live and (d) drug-loaded fiber 76% dead/24% live.
