# Supplementary material for: A Portable Device for the Generation of Drug-Loaded Three-Compartmental Fibers Containing Metronidazole and Iodine for Topical Application
Source: Pharmaceutics. 2020 Apr 18;12(4):373. doi: 10.3390/pharmaceutics12040373 (PMC7238112; doi:10.3390/pharmaceutics12040373)
Supplement: Supplementary file 1 [file pharmaceutics-12-00373-s001.pdf]

pharmaceutics

Supplementary Materials: A Portable Device for the Generation of Drug-Loaded Three-Compartmental Fibers Containing Metronidazole and Iodine for Topical Application

Francis Brako, Chaojie Luo, Rupy Kaur Matharu, Lena Ciric, Anthony Harker, Mohan Edirisinghe and Duncan Q. M. Craig

1. Making the Three-Layered Drug-Loaded Fibers

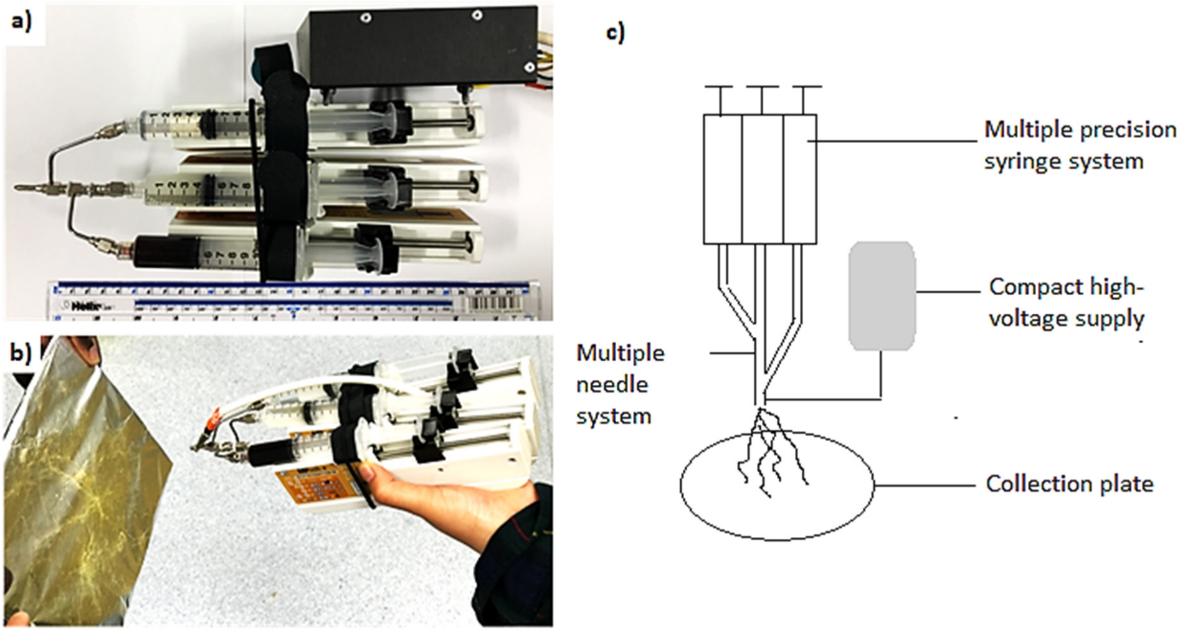

Figure S1. (**a**) Assembling various components of the portable electrospinning device; (**b**) the device in operation, producing yellowish metronidazole/iodine loaded fibers; and (**c**) a schematic representation of the device setup.

2. Mathematical Relationships between the Actual Drug Released from the Delivery Systems and the Sampled Drugs after Movement across A Dialysis Membrane in the Two-Chamber System Used to Compare the Release of Drugs from the Samples Used in the Study

To compare the release of drug from the trilayered fibersand creams, a setup comprising of the delivery system enclosed within a dialysis membrane in a volume of 5 ml and further immersed in a liquid with a volume of 45 ml (outer chamber) was used, from which sampling was taken from the outer chamber to determine the amount of drug released. This was to ensure that the delivery systems being studied were totally immersed within the dissolution media as both systems had tendencies to float, which could disrupt the continuous release of the drug into the media.

In this two-chamber setup, the sampling for the analysis of the drug release was possible from the outer chamber, but as the drug was released into the inner chamber enclosed by a dialysis membrane, it was necessary to establish the relationship between the sampled release and actual drug released to determine if the sampled drug reflected the actual amount of drug released. For a more robust comparison between the drug released from creams and the trilayered fibers, it was necessary to establish that regardless of the difference in the diffusion coefficient, which was certain to be the case for creams and fibers, the amount of drug sampled from the outer chamber for analysis maintained its relationship with the actual amount of drug released within the dialysis membrane.

To determine this relationship, some assumptions and modelling of the experimental data from the study were made. These were essential to establish an accurate comparison of the drug release from the trilayered fibers and creams. It was necessary to assume a similar geometry for both delivery systems. A sphere was assumed, as that was the closest shape to a rounded mass of fiber or cream placed in the dialysis tube of the study; hence, spherical symmetry was assumed throughout. It was also necessary to assume that the flow around the delivery system was sufficiently free for the concentration of the drug dissolved in the liquid inside the dialysis membrane, $c_2(t)$, to be spatially uniform; and that the same was true of the concentration in the outer liquid, $c_4(t)$. In other words, the drug was distributed through the liquid by a rapid combination of diffusion and convection. The concentrations in the delivery system $c_1(r, t)$ and in the membrane $c_3(r, t)$, on the other hand, depended on both time and position, and it was diffusion in those media that was assumed to control the release rates. The active ingredient was assumed to be initially uniformly distributed in the delivery system with a radius a; and it diffused with the diffusion coefficient D_p towards the liquid contained in the dialysis membrane.

Furthermore, to vary the volume of the delivery system for the purposes of generating a trend of drug release, the system was assumed to be made of a number of discrete particles N_p, and hence with a total volume of $N_p v_p$, also equal to $N_p \frac{4}{3}\pi a^3$. Then, let the volume of the liquid within the membrane be wV_p, the dialysis membrane extending from r_{m1} to r_{m2} and the drug diffusing through it with the diffusion coefficient D_m into the outer liquid of volume vwV_p with the partition coefficients K_{ij} (K_{12}, K_{23} *and* K_{34} for the partitioning coefficients from the delivery system into the inner chamber, inner chamber into the membrane and membrane into the outer chamber, respectively) at the solid-liquid interface. During the experiments, the outer liquid was sampled and replaced with drug-free liquid so that the fraction removed in the time Δt was given as $\alpha\Delta t$.

With all these assumptions in place, the concentrations of the drug in the delivery system (c_1), in the liquid enclosed in the membrane (c_2), inside the membrane (c_3) and in the outer chamber where sampling occurred (c_4) could be determined by solving Equations (1) to (11) as follows:

$$\frac{\partial(rc_1\ (r,t))}{\partial t} = D_p \frac{\partial^2\ (rc_1\ (r,t))}{\partial r^2} \qquad 0 \le r \le a \tag{1}$$

$$c_1\ (r,0) = c_0 \qquad r \le 0 \le a \tag{2}$$

$$c_2(t) = K_{12} c_1(a,t) \tag{3}$$

$$c_2(0) = 0 \tag{4}$$

$$\frac{dc_2(t)}{dt} = -4\pi[a^2 D_p \frac{\partial c_1(r,t)}{\partial r} \mid \quad r = a\ \frac{1}{wv_p} - r_{m1}^2 D_m \frac{\partial c_3(r,t)}{\partial r} \mid \quad r = r_{m1}\ \frac{1}{N_p w v_p}] \tag{5}$$

$$c_3(r,0) = 0 \qquad r_{m1} \le r \le r_{m2} \tag{6}$$

$$c_3(r_{m1},t) = K_{23} c_2(t) \tag{7}$$

$$\frac{\partial(rc_3\ (r,t))}{\partial t} = D_m \frac{\partial^2\ (rc_3\ (r,t))}{\partial r^2} \qquad r_{m1} \le r \le r_{m2} \tag{8}$$

$$c_4(t) = K_{34} c_3(r_{m2},t) \tag{9}$$

$$c_4(0) = 0 \tag{10}$$

$$\frac{dc_4(t)}{dt} = -4\pi r_{m2}^2\ D_m \frac{\partial c_3(r,t)}{\partial r} \mid \quad r = r_{m2}\ \frac{1}{N_p v w v_p} - r_{m1}^2 \alpha c_4(t) \mid \tag{11}$$

In principle, the equations can be solved by Laplace transform techniques, but the resulting expressions are somewhat unwieldy. Furthermore, the constant sample rate α is not truly representative of the finite samples taken at discrete time intervals in the experimental investigation. We have therefore adopted a numerical procedure based on a forward Euler scheme [1], in which the concentration in the delivery system is defined as N_1 discrete units distributed over regularly-spaced

radial grids at Δr_1 intervals; and for the drug concentration in the membrane as N_3 units over Δr_3 intervals.

That gives the following Equations (12) to (14):

$$c_1(i\Delta r_1, n\Delta t) = c_{1,i,n}, 0 \le i \le N_1 \quad (12)$$

$$c_3(r_{m1} + i\Delta r_3, n\Delta t) = c_{3,i,n}, 1 \le i \le N_3 \quad (13)$$

$$c_{s,i,n+1} = c_{s,i,n} + \Delta t \frac{dc_{s,i,n}}{dt} \quad (14)$$

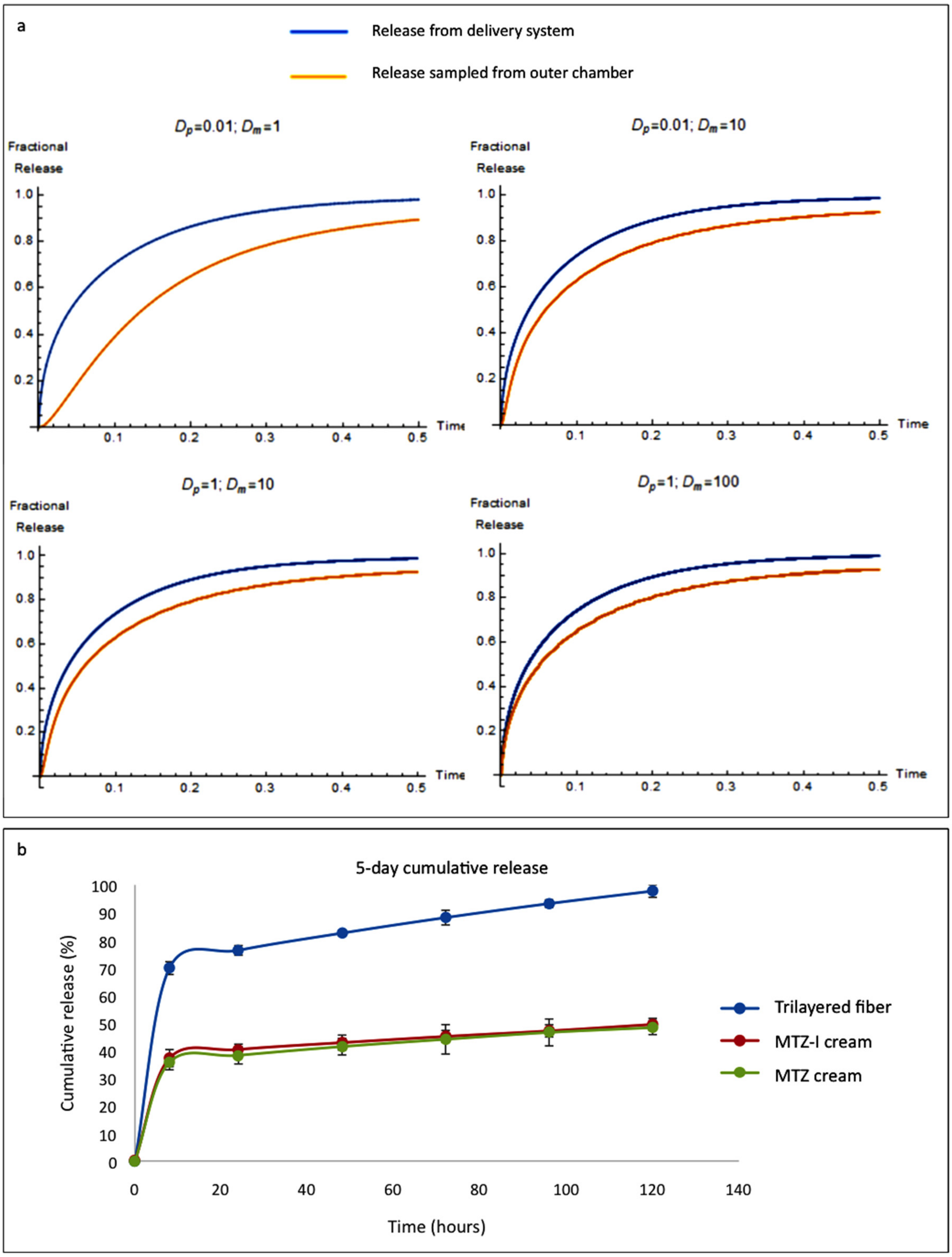

Figure S2. (**a**) Fractional release from the delivery systems (blue) and into the outer liquid (orange) as a function of time for scenarios with varying diffusion coefficients from the delivery system (D_p) and through the dialysis membrane (D_m). (**b**) Cumulative percentage of drug released from the trilayered fibersystems and cream formulations obtained during the study over a five-day period.

Using a second-order finite difference approximation to the spatial derivatives, with the usual special form at r = 0 [2] and coding these expressions in Mathematica®, one-sided difference expressions were used to obtain the gradients at the solid-liquid interfaces. Forty radial divisions in the assumed spherical delivery systems were found: the time-step Δt was chosen to ensure the stability of the adopted Euler scheme. For illustrative purposes, we have taken all partitioning coefficients *Kij* to be 1 and the membrane thickness to be 0.1 mm.

Drug release trends based on different diffusion coefficients (D_p *and* D_m) were then generated based on the expressions illustrated above to investigate if the amount of drug sampled from the outer chamber reflected that which was actually being released from the delivery system into the inner chamber. The release profiles obtained are shown in Figure S1.

It is clear that the presence of a membrane between the drug-laden particles and the sampled volume can have an effect on the observed drug release rate. There are, however, no clearly identifiable differences in the overall shapes of the curves. In light of these assumptions and the mathematical definition of likely scenarios in our experimental setup, all that can be firmly concluded is that the release of the drug from the examined delivery systems occurred in trends not different from those measured by sampling the liquid in the outer chamber. Therefore, the amount of drug sampled from the outer chamber, which was a direct function of the actual drug released from the delivery system, was used subsequently to compare the performance of the trilayered fibers and creams formulated to contain similar amounts of active drugs.

3. Antibacterial Studies

3.1. LB Agar Plates Assessment of Fibersand Creams

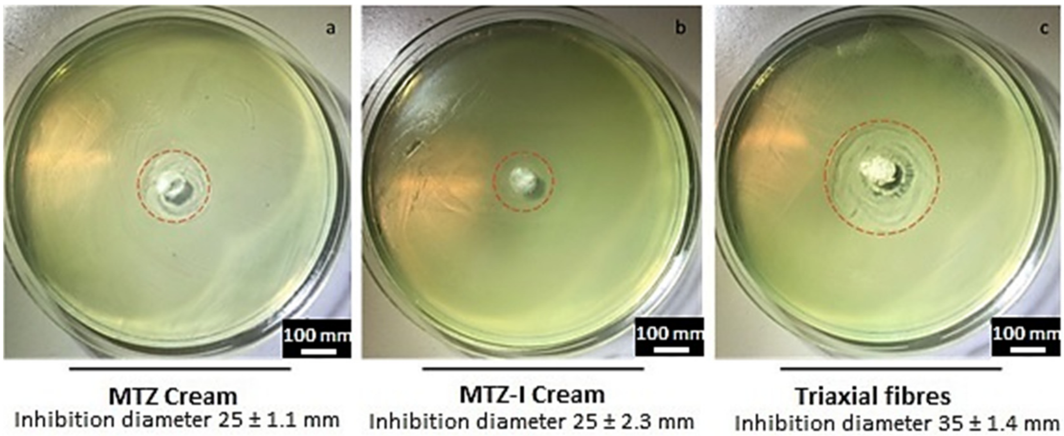

Figure S3. Photographs of the results showing the growth inhibition of *P. aeruginosa* following the 24-hour incubation of the bacterial cells on LB agar plates with: (**a**) MTZ cream, (**b**) MTZ-I cream, and (**c**) triaxial fibers.

3.2. Flow cytometry

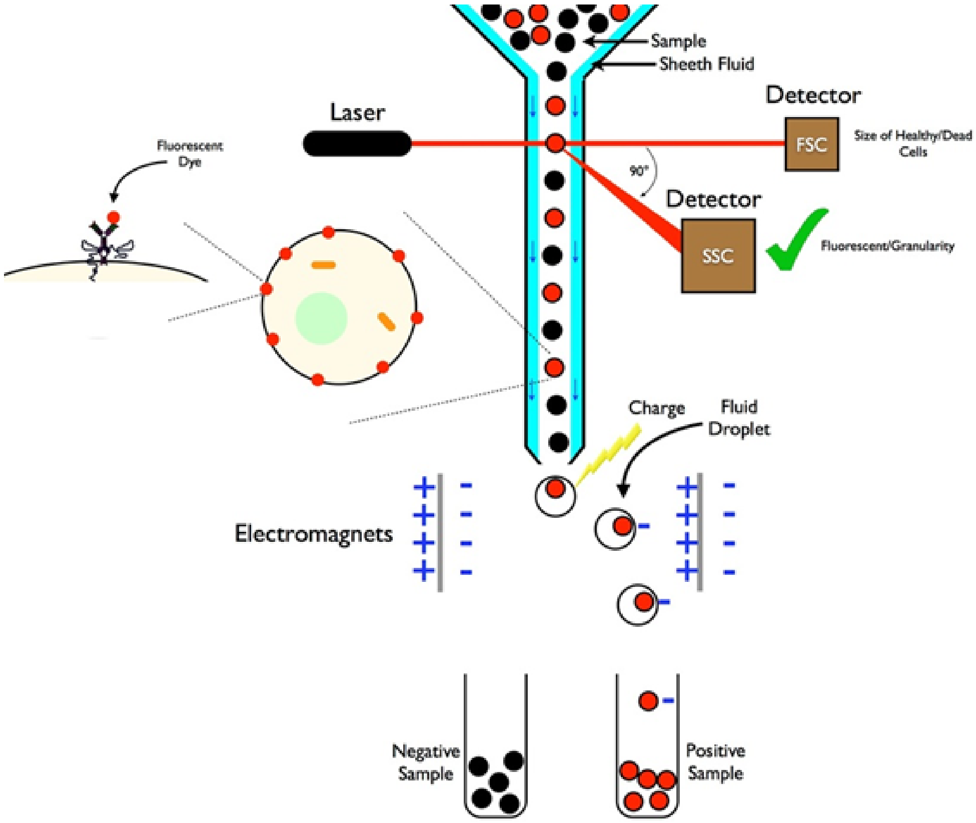

Figure S4. A schematic representation of cell sorting using flow cytometry [Image source: Sari Sabban ©2011 / CC BY-SA (https://creativecommons.org/licenses/by-sa/3.0)].

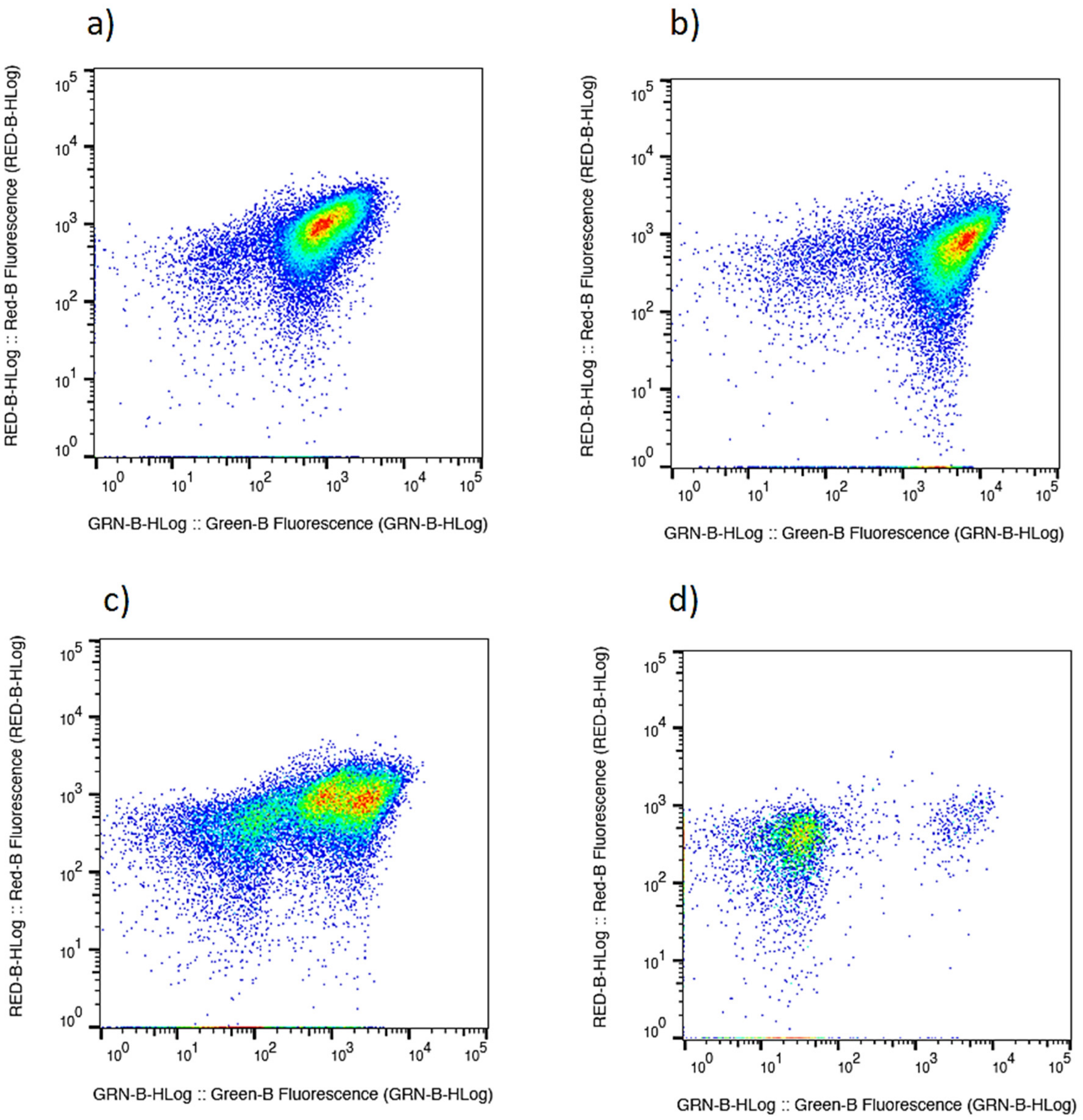

Figure S5. Dead/live cell count by flow cytometry of cell suspensions incubated for 24 hours for (**a**) control (without cream or fiber) 4% dead/96% live, (**b**) plain fibers without drug 3% dead/97% live, (**c**) drug-loaded cream 59% dead/41% live and (**d**) drug-loaded fiber 76% dead/24% live.

References

[1] J. C. Butcher, *The Numerical Analysis of Ordinary Differential Equations. Runge-Kutta Methods and General Linear Methods*, John Wiley, Chichester 1987.

[2] J. Crank, *The Mathematics of Diffusion*, Oxford University Press, Oxford 1956.
.49%. Found: C, 57.01; H, 5.75; N, 14.23%

© 2020 by the authors. Licensee MDPI, Basel, Switzerland. This article is an open access article distributed under the terms and conditions of the Creative Commons Attribution (CC BY) license (http://creativecommons.org/licenses/by/4.0/).
